# Mitigating aerosol contamination: strategies for contamination-free scaling

**DOI:** 10.3205/dgkh000604

**Published:** 2025-12-05

**Authors:** Arathi Shenoy, Nina Shenoy, Chandra Kolluru Subhash

**Affiliations:** 1 Nitte (Deemed to be University), AB Shetty Memorial Institute of Dental Sciences (ABSMIDS), Department of Periodontology, Mangalore, India

**Keywords:** ultrasonic scaling, aerosol contamination, infection control, evacuation, air decontamination

## Abstract

**Introduction:**

Ultrasonic scaling has transformed periodontal therapy, delivering effective plaque and calculus removal. However, it also introduces risks through the generation of aerosols and splatter, which can transmit infectious agents. This review evaluates strategies to mitigate aerosol contamination in modern dental practice.

**Results:**

Pre-, intra-, and post procedural measures can significantly minimize contamination. Adequate ventilation, appropriate coolant selection, and preprocedural mouthrinses limit aerosol generation. Personal protective equipment reduces exposure, while high-volume evacuators (HVEs) effectively capture aerosols. After the procedure, thorough instrument reprocessing and surface disinfection, along with the use of high-efficiency particulate air filters (HEPA) and UV irradiation, are essential for air decontamination.

**Conclusion:**

Ongoing research continues to enhance control protocols. Implementing these measures effectively safeguards both patients and dental personnel, particularly in light of respiratory infections such as flu.

## Zusammenfassung

**Einführung:**

Ultraschall-Scaling hat die Parodontaltherapie durch die effektive Entfernung von Plaque und Zahnstein verändert, birgt aber auch Risiken durch Aerosole und Spritzer, die Krankheitserreger übertragen können. In dieser Übersicht werden Strategien zur Verringerung der Aerosolkontamination in modernen Zahnarztpraxen bewertet.

**Ergebnisse:**

Maßnahmen vor, während und nach dem Ultraschall-Scaling zielen darauf ab, die Kontamination zu minimieren. Belüftung, Auswahl des Kühlmittels und Spülungen vor der Behandlung verringern die Aerosolbildung. Persönliche Schutzausrüstung verringert die Exposition, während hochvolumige Absauggeräte (HVEs) Aerosole wirksam entfernen. Nach dem Scaling sind die Aufbereitung der Instrumente und die Oberflächendesinfektion unerlässlich. HEPA-Filter und UV-Bestrahlung unterstützen die Dekontamimation der Luft.

**Schlussfolgerung:**

Die Forschung treibt die Entwicklung von Protokollen zur Infektionskontrolle weiter voran. Die wirksame Umsetzung dieser Maßnahmen gewährleistet die Sicherheit von Patienten und Personal im zahnärztlichen Umfeld, insbesondere als Schutz vor respiratorischen Infektionen wie bei der Virusgrippe.

## Introduction

Maintaining good oral health is essential for overall well-being. Regular oral prophylaxis is a fundamental aspect of preventive dentistry, crucial for achieving optimal oral health. In contemporary dentistry, ultrasonic scaling has transformed periodontal therapy by providing efficient removal of plaque and calculus. However, it also raises concerns regarding the generation of aerosols, which can pose a potential risk for the transmission of infectious diseases. 

In a periodontal operatory setting, infections can spread through direct contact with blood and oral fluids, as well as indirectly via contaminated instruments, objects, or surfaces [1]. Among dental procedures that generate visible aerosol clouds, ultrasonic scaling is identified as the primary source, followed by high-speed handpieces, air-abrasion units, and air-water syringes [2].

The prolonged presence of aerosols in the air increases the risk for transmitting respiratory infections, since small particles containing infectious agents can persist even after droplet evaporation. This ongoing exposure poses significant risks to both dental professionals and patients [3]. 

This article aims to review and analyze measures and strategies to minimize aerosol contamination throughout the various stages of ultrasonic scaling in contemporary dental practices.

## Contamination risk in periodontal practice

Direct contact with blood, saliva, and other oral fluids may occur during the scaling procedure or during other periodontal interventions, creating a risk of transmission of pathogens such as hepatitis B or C if proper infection control protocols are not followed [4]. Indirect transmission can take place through contact with contaminated instruments or surfaces within the operatory. 

Airborne transmission occurs through acts such as coughing, sneezing, or talking that generate respiratory droplets. Airborne infection risks also stem from inhaling contaminated aerosols produced during dental procedures [5]. Examples of pathogens that can be transmitted via aerosols generated during dental procedures include *Staphylococcus aureus*, influenza virus, hepatitis B and C viruses and, SARS-CoV-2 virus [6], as listed in Table 1 (Tab. 1).

The ultrasonic scaler operates at a frequency of 25,000 to 30,000 cycles per second, which can cause the instrument to heat up. This heat build-up requires continuous application of coolant [7]. When the vibrating tip of the ultrasonic scaler comes into contact with the coolant, aerosol and splatter are generated. Micik et al. [8] described aerosol as a mixture of liquid or solid particles suspended in air, with sizes ranging from 5 µm to 50 µm. These particles can evaporate into droplet nuclei that serves as reservoirs for microorganisms [3]. They may contain blood, saliva, bacteria, and respiratory or oropharyngeal secretions [9]. These minute particles can penetrate and become lodged in the narrow passages of the lungs, posing a health risk [3]. 

Air-water syringes also have the potential to produce aerosols. It has been stated that the level of bacterial contamination from air-water syringes is nearly equal to that from ultrasonic scalers [3]. A study by Inger et al. [10] found that non-disposable air-water syringe tips were more contaminated than disposable tips after sterilization. Although disposable tips may help minimize the risk of cross-infection, they raise concerns regarding sustainability. If used, opting for biodegradable alternatives would be more environmentally responsible.

## SARS-CoV-2 and aerosols

The COVID-19 pandemic has highlighted potential risks associated with procedures that generate aerosols. Aerosols produced during ultrasonic scaling also contain SARS-CoV-2 [11]. The World Health Organization has stated that the primary mode of transmission of the coronavirus is through saliva droplets and nasal discharge [12]. Studies have shown a higher prevalence of COVID-19 antibodies among dentists than in the general population [13]. The replication capacity of the virus in aerosols remains a topic of ongoing research. While some studies have shown a rapid decline in viral viability after aerosolization, others suggest that the virus may remain viable for extended periods under certain environmental conditions [12]. Given the critical importance of protecting both patients and dental professionals, the following sections will outline essential precautions to minimize aerosol contamination. 

## Dental unit water lines (DUWLs)

Biofilms that develop within DUWLs can harbor bacteria, including opportunistic pathogens such as *Pseudomonas aeruginosa*, *Legionella pneumophila*, and nontuberculous mycobacteria [14]. Dental units without proper treatment cannot consistently produce water that meets the drinking water standards, making it essential to comply with CDC guidelines [15]. The management of DUWLs will be addressed in subsequent sections.

It is crucial to manage contamination effectively, even for relatively simple procedures like ultrasonic scaling. Stringent measures should be implemented before, during, and after the procedure. Table 2 (Tab. 2) illustrates the stepwise contamination control protocols for ultrasonic scaling.

## Measures to be taken before initiating ultrasonic scaling

### Pre-procedural screening

A clinical examination of patients before ultrasonic scaling is essential for infection control, especially for identifying those with respiratory or contagious diseases who are at higher risk of transmitting aerosols. This examination helps in formulating customized treatment plans, which may involve implementing additional infection control protocols or delaying treatment. It is crucial to record a thorough medical and dental history and conduct a detailed oral examination before initiating ultrasonic scaling [16]. 

In addition to clinical diagnosis, evaluating prognosis and disease risk is important to determine whether patients are healthy, experiencing an active infection, or in remission. This assessment is key to ensuring that appropriate precautions are taken [17]. 

### Ensuring well-ventilated operatory

Effective ventilation is essential for managing the aerosols produced during dental procedures. Airflow patterns and particle size are crucial in determining how deeply these particles can enter the lungs. Smaller particles, particularly those less than 2 microns in size, are significantly affected by Brownian motion, which is the erratic movement of tiny particles suspended in a medium (e.g., air). This random motion allows these small particles to evade respiratory defenses and settle deep within the alveoli, posing potential health risks [18]. 

To enhance air circulation and promote laminar airflow, several measures can be taken: keeping windows open, refraining from use of a ceiling or pedestal fans, and utilizing exhaust fans. Laminar airflow refers to a situation where air flows uniformly in one direction at a consistent speed, with minimal mixing or crossover of air streams. A study has shown that laminar airflow can reduce spread of bacteria by 99.6%. Therefore, it is essential to adopt new strategies while maintaining proper ventilation in light of modern technology [19].

### Water coolant considerations

Water coolants are directed towards the tip of magnetostrictive ultrasonic scalers to mitigate heat generation and prevent potential injury to pulp and periodontal tissue. Distilled water remains the preferred coolant in the ultrasonic water system due to its ready availability, affordability, and time-tested use. Various studies have explored alternative coolants, yielding various results. Jawade et al. [20] compared the effectiveness of ultrasonic coolants such as povidone-iodine and chlorhexidine gluconate in reducing aerosol production by counting the number of colony-forming units on agar plates exposed to aerosols from different coolants. It was concluded that chlorhexidine gluconate is more effective in reducing dental aerosols when compared to povidone and distilled water. Additionally, alternative ultrasonic coolant sources, e.g., essential oils cinnamon extract, have shown promise in reducing aerosol generation, resulting in fewer colony-forming units on agar plates [20], [21]. 

Researchers have proposed incorporating food-grade thickening agent into the coolants [22]. These additives can modify the physical properties of water, offering a cost-effective solution to eliminate aerosol production without major equipment or procedural changes. This approach is theoretically more effective in preventing aerosol exposure to dental personnel and patients, aligning with CDC’s hierarchy of controls [23]. A study by Farah et al. [24] assessed the efficacy of polyacrylic acid, xanthan gum, and carboxymethyl cellulose as thickeners, showing significantly smaller areas over which aerosols, splatter, and droplet contamination can disperse.

### Pre-procedural rinse

It is recommended for patients to use a mouthrinse 2 to 40 minutes before starting ultrasonic scaling to reduce the risk of aerosol contamination. This rinse typically employs an antimicrobial or antiseptic solution to reduce oral microbial count, minimizing bacterial spread during the procedure.

Commonly used solutions used for pre-procedural rinse include:

- Chlorhexidine gluconate, which is a widely used gold standard rinse with broad-spectrum antimicrobial properties. A systematic review by Koletsi et al. [25] showed that tempered chlorhexidine digluconate (CHG) 0.2% rinse showed the strongest effect, with a significant decrease in bacterial load compared to a control rinse. CHG 0.2% without temperature control and chlorine dioxide rinses also showed similar trends of reducing bacteria, though to a slightly lesser extent.
- Povidone iodine is another antiseptic solution that is effective against a wide range of microorganisms. In clinical practice, a 30- or 60-s rinse with 0.5% or 1% povidone-iodine may offer a potential benefit [26].
- A diluted solution of 1.5% hydrogen peroxide can also be used as a pre-procedural rinse. It exerts therapeutic effects by releasing oxygen, which rapidly eliminates obligate anaerobes found in oral infections. It can also be used in combination with 0.2% CHG [27].
- 0.05% cetylpyridinium chloride has proven to be effective in controlling aerosol contamination. It acts primarily by penetrating the bacterial cell membrane, causing leakage of cell components, disrupting bacterial metabolism, and ultimately leading to cell death [28].
- Essential oils have been used since the 19^th^ century, and mouthwashes containing herbal extracts or essential oils have been employed. Commercially available mouthrinses containing thymol, menthol, eucalyptol, and methyl salicylate can be used. The antimicrobial properties of essential oils, coupled with their potential to enhance patient compliance make them attractive for minimizing the spread of infectious agents through aerosols [29].

### PPE

PPE must be donned for treating every patient, regardless of their infection status, as a standard universal precaution. The consistent use of PPE ensures protection against asymptomatic carriers and minimizes the risk of cross contamination. The universal application of PPE is a cornerstone of infection control protocols in dental practice.

Donning personal protective equipment is essential for both patients and dental professionals. Water-resistant drapes for patients can decrease the likelihood of aerosol particles settling on clothing. Table 3 (Tab. 3) enlists recommended PPE for dental professionals.

After patient treatment, all exterior surfaces of PPE should be treated as potentially contaminated. The use of disposable plastic covers on surfaces and other areas exposed to aerosols (for instance, light handles and chair controls) should be considered.

## Measures to be taken during ultrasonic scaling

### Clinician and patient considerations

Clinicians should prioritize patient positioning and ergonomics to minimize the dispersion of aerosols and splatter during procedures. A systematic review by Johnson et al. [30] found that the highest levels of contamination occurred around the dentist's head and the patient's mouth and chest during ultrasonic scaling. This highlights the need for proper ergonomics and positioning to prevent water splashing and aerosol dispersion, aiming to contain it and prevent it reaching the patient’s face or surroundings. 

Research has shown that the placement of the handpiece within the dental arch impacts splatter production. When water spray is directed closer to the oral opening – such as near the maxillary front teeth – there is a greater likelihood of water exiting the mouth rather than adhering to oral surfaces nearby [31]. To minimize aerosols and splatter, it is essential to maintain control over the ultrasonic handpiece and avoiding excessive pressure on the scaler tip.

The patient can be positioned supine with head turned sideways and slightly tilted downwards to pool liquid around the corner of the mouth for easy suction and evacuation [32]. 

Water containment prevents the escape of water in the form of aerosols. One alternative technique for chieving this is the cupping technique. This technique uses patient’s lip and cheeks as fluid deflecting barrier. Pulling lip upwards and outwards for mandibular anterior teeth and holding the cheek away for posterior teeth creates barriers to deflect water back into mouth, aiding in efficient evacuation and controlling aerosols dispersion [33]. 

### Power adjustments

In terms of how well the treatment works, using the ultrasonic scaler at half power is as effective as using it at full power [33]. Adjusting the power changes only the amplitude, not the frequency. Hence lower power means lower vibration strength. Medium to high power settings generally aerosolize the water, as the higher amplitude causes water droplets to atomize. By choosing a lower power setting, the amount of water can be reduced to a drip rather than a spray. This ultimately results in the generation of less aerosol during the procedure [17]. 

### Minimizing water spray

Finding the right balance is the key, and it lies in determining the optimal water flow that provides adequate cooling and debris removal while minimizing aerosols. It is advisable to use short bursts of water spray when necessary for visibility, which helps reduce overall exposure time to aerosols [34]. 

### HVEs

HVEs are suction devices that can remove up to 100 cubic feet of air per minute. The American Dental Association recommends HVEs over saliva ejectors for managing pooled fluids, as the latter are less effective in reducing aerosols clouds due to their small opening and lower air removal capacity [35]. HVEs can reduce aerosols and splatter by 93–96% by efficiently drawing in air when positioned near the patient's mouth. While test outcomes may vary, they generally demonstrate a reduction of aerosols by 90.8% [36]. Clinicians should carefully monitor technical specifications such as power and airflow volume settings to ensure effectiveness. It is recommended to maintain a distance of 6 to 15 mm from the active ultrasonic tip when using HVEs. In multi-chair clinics, shared suction systems may lead to reduced volume and pressure. Ergonomic considerations are also important, as the weight and handling of devices can significantly affect aerosol management [37].

Clinicians should monitor technical specifications such as power and airflow volume settings to ensure effectiveness. Clinicians should maintain a distance of 6 to 15 mm from the active ultrasonic tip when using HVE devices. In multi-chair clinics, shared suction systems may lead to reduced volume and pressure. Ergonomic considerations are crucial, as device weight and handling can affect aerosol management [36]. 

## Post-scaling measures

After ultrasonic scaling, proper teprocessing of instruments and maintenance of equipment are crucial to controlling infection by eliminating pathogens. This practice helps prevents cross-contamination, reduces the risk of aerosols and splatter, and ensures the safety of both patients and dentists while maintaining a hygienic dental environment.

Aerosols can settle on objects and surfaces near the dental chair, making it essential to clean and disinfect any contaminated surfaces. Disinfectants are available as aerosols, sprays and wipes which contain powerful chemicals. Agents such as benzalkonium chloride can be effective when used in sprays and wipes. Other potent disinfectants are ethanol, propan-2-ol, sodium hypochlorite, and glutaral. Wipes are particularly suitable for non-critical surfaces such as instrument trays and side tables. The primary objective of these substances is to disrupt bacterial cell structures, leading to eventual lysis of the microorganisms [38]. 

## High-efficiency particulate air filters (HEPA)

Using HEPA can significantly reduce aerosol contamination after ultrasonic scaling procedures. A HEPA captures at least 99.7% of particles that are ≥0.3 microns in size, which implies that out of every 10,000 particles passing through the filter, only three particles escape [38], [39]. Due to its high efficiency, its use in dental operatories is well accepted.

The filter medium of HEPA consists of a dense mat of randomly arranged fibers. This arrangement creates a maze-like structure that forces air to follow a tortuous path as it passes through the filter. This intricate design enhances the likelihood of particles colliding with the fibers and being captured [40]. 

## Other measures

Ultraviolet (UV) irradiation is also employed for air decontamination. UV lamps emit radiation that disrupts the DNA of bacteria and viruses, rendering them sterile and unable to reproduce. UV-C light, with wavelengths between 250 nm and 265 nm, is typically used for this purpose.

The management DUWLs is essential to prevent microbial growth. At the end of each day, it is important to purge these lines by running water through them to flush out any accumulated biofilm, bacteria, or contaminants. Flushing DUWLs with disinfecting agents yields better results [41]. 

Chemical agents used for disinfection include chlorine-based disinfectants, e.g., chlorine dioxide and chlorine hypochlorite, peroxide-based disinfectants, e.g., hydrogen peroxide, as well as quaternary ammonium compounds.

## Recent advances in management of aerosols

In response to the challenges associated with HVEs, which require personnel to hold, position, and manipulate the device, extraoral local extractors (ELEs) have emerged as a viable alternative. Operating on a similar principle as saliva ejectors or HVEs, ELEs distinguish themselves through a higher flow rate and an extended working distance. These devices, introduced and marketed during the onset of the COVID-19 pandemic, alleviate the need for an assistant. The effectiveness of local extractors (LEs) surpasses that of traditional clinical aerosol-management strategies, and have demonstrated their efficacy without requiring additional personnel. It is evident that the proper positioning of the nozzle and management of airflow are crucial for optimal performance, yet this can be achieved without hindering the work position of dental hygienists. Notably, ELEs can be designed to remain fixed in position, eliminating the necessity for an extra assistant to operate them. This innovation presents a practical solution to enhance dental hygiene practices, especially in scenarios where traditional HVEs may pose challenges [42].

In a study conducted by Hidalgo et al. [43], the efficiency of an ultrasonic insert with a focused coolant spray was evaluated. This device was designed to enable the cooling water to exit through the scaler tip, intending to focus the water spray. The focusing aimed to reduce aerosol production, consequently minimizing contamination. The device's design did not achieve its intended actions. To utilize this efficiently designed device, it is essential to conduct in-depth research and ensure a comprehensive understanding of its functionality.

The Clara Mask, an innovative, PPE product (pending NIOSH approval) is a reusable, clear mask respirator designed for passive air filtration. It features replaceable 99.97% HEPA filter ports for inhalation only or a different model with inhalation and exhalation ports. This sustainable solution aims to reduce waste generated by disposable masks and offers cost saving through reusability. Its development in the post-COVID-19 era signifies advancements in PPE design and functionality [44]. 

Antimicrobial nanocoating, when applied to surfaces, can inhibit bacterial attachment and the development of biofilms, providing a localized and long-lasting antimicrobial resistance. This is especially useful for medical implants and frequently touched surfaces in dental settings. Incorporating nanomaterials into aerosol-capture systems (e.g., HVE tips and air purifiers) can further enhance microbial trapping and inactivation. Additionally, pre-procedural mouthrinses containing nanoparticles could lower the initial oral microbial load, thus decreasing aerosolized microorganisms, although further research is required to confirm safety and effectiveness [45].

## Conclusion

Effective management of aerosol contamination in ultrasonic scaling is of paramount importance for ensuring patient and staff safety in dental practice. Through comprehensive preprocedural screenings, ventilation optimization, proper coolant selection and diligent use of PPEs, risks can be significantly mitigated. Moreover, advancements such as HVEs and HEPA offer promising avenues for further reducing aerosol transmission. 

## Notes

### Authors’ ORCIDs

- Shenoy A: https://orcid.org/0009-0008-8592-7580
- Shenoy N: https://orcid.org/0000-0002-1189-6901
- Subhash CK: https://orcid.org/0009-0008-6118-3716

### Funding

None. 

### Competing interests

The authors declare that they have no competing interests.

## Figures and Tables

**Table 1:**
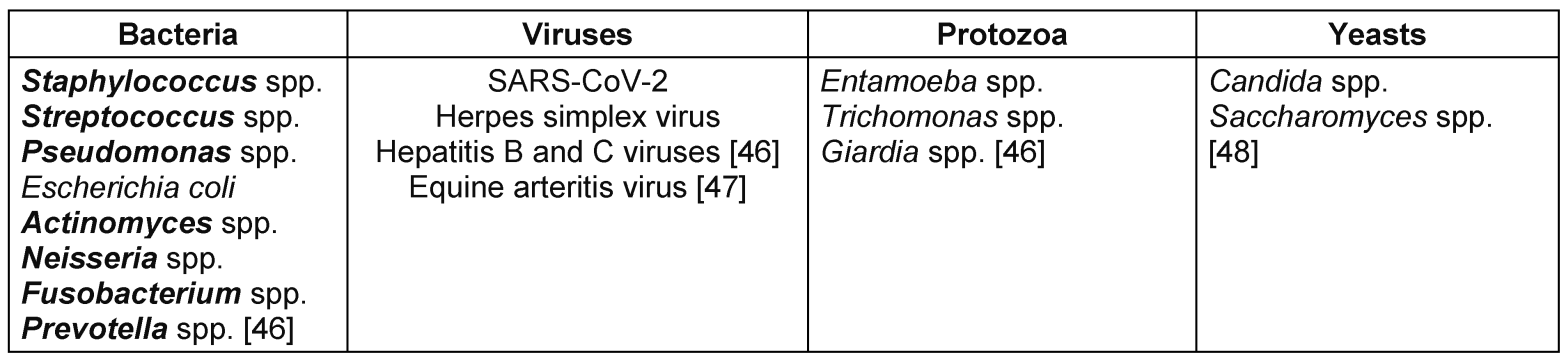
Pathogens transmissible via aerosols during ultrasonic scaling

**Table 2:**
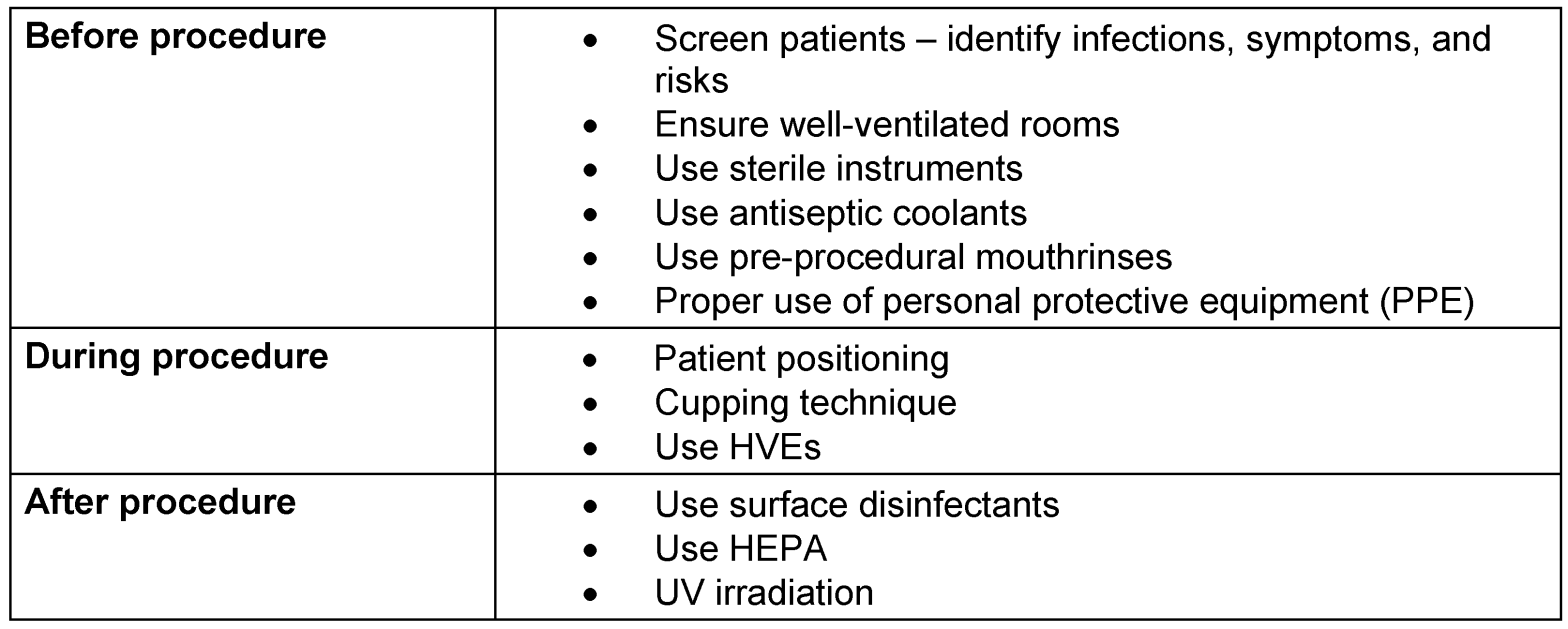
Stepwise contamination control protocols during ultrasonic scaling.

**Table 3:**
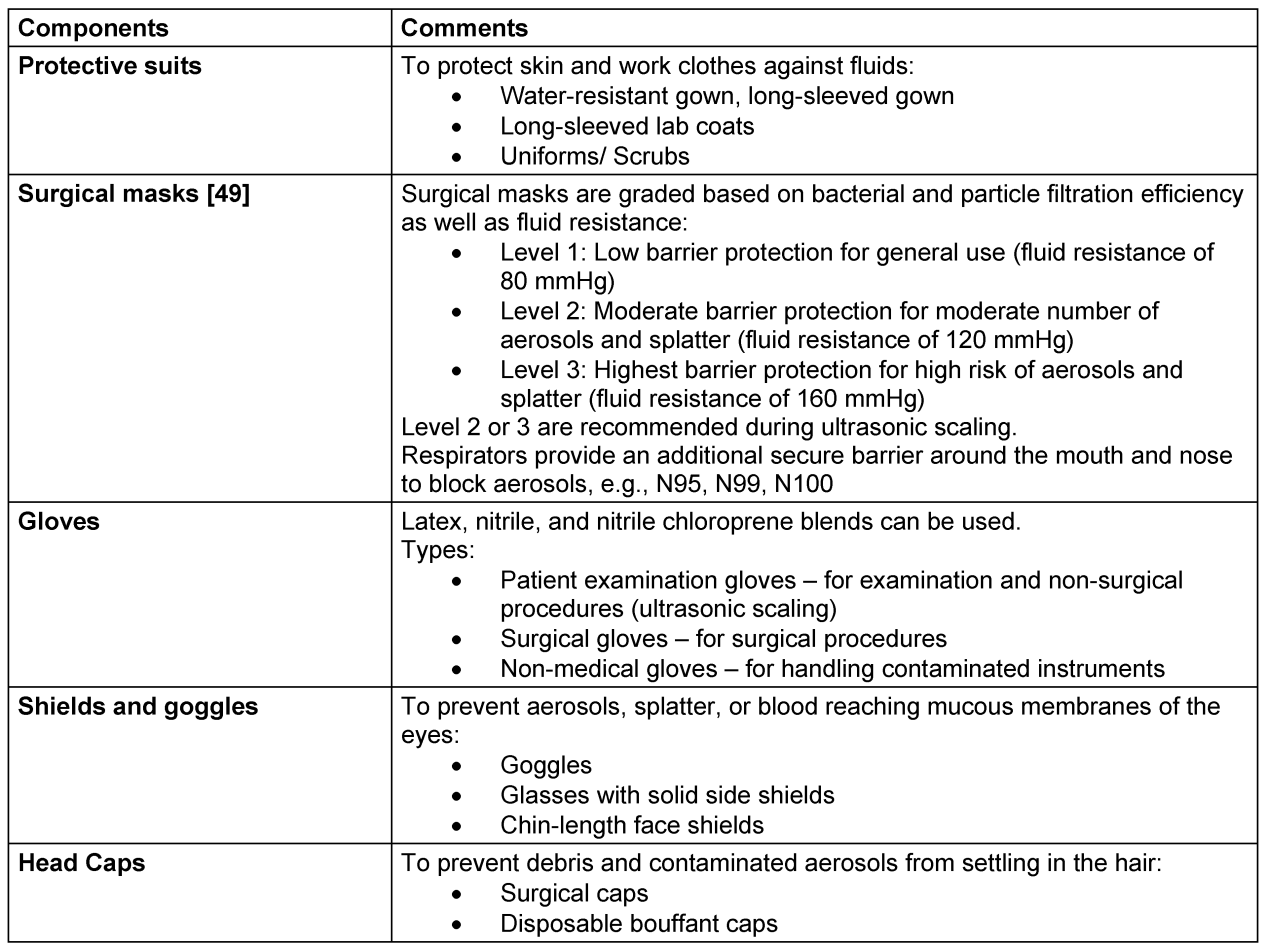
Components of recommended PPE for ultrasonic scaling
